# *CACNA1C* Risk Variant and Amygdala Activity in Bipolar Disorder, Schizophrenia and Healthy Controls

**DOI:** 10.1371/journal.pone.0056970

**Published:** 2013-02-20

**Authors:** Martin Tesli, Kristina C. Skatun, Olga Therese Ousdal, Andrew Anand Brown, Christian Thoresen, Ingrid Agartz, Ingrid Melle, Srdjan Djurovic, Jimmy Jensen, Ole A. Andreassen

**Affiliations:** 1 Institute of Clinical Medicine, University of Oslo, Oslo, Norway; 2 Division of Mental Health and Addiction, Oslo University Hospital, Oslo, Norway; 3 Department of Medical Genetics, Oslo University Hospital, Oslo, Norway; 4 HUBIN Project, Psychiatry Section, Department of Clinical Neuroscience, Karolinska Institutet and Hospital, Stockholm, Sweden; 5 Department of Psychiatric Research, Diakonhjemmet Hospital, Oslo, Norway; 6 Department of Psychiatry and Psychotherapy, Charité Universitätsmedizin, Berlin, Germany; 7 Wellcome Trust Sanger Institute, Wellcome Trust Genome Campus, Hinxton, United Kingdom; University of Minnesota, United States of America

## Abstract

**Objectives:**

Several genetic studies have implicated the *CACNA1C* SNP rs1006737 in bipolar disorder (BD) and schizophrenia (SZ) pathology. This polymorphism was recently found associated with increased amygdala activity in healthy controls and patients with BD. We performed a functional Magnetic Resonance Imaging (fMRI) study in a sample of BD and SZ cases and healthy controls to test for altered amygdala activity in carriers of the rs1006737 risk allele (AA/AG), and to investigate if there were differences across the diagnostic groups.

**Methods:**

Rs1006737 was genotyped in 250 individuals (N = 66 BD, 61 SZ and 123 healthy controls), all of Northern European origin, who underwent an fMRI negative faces matching task. Statistical tests were performed with a model correcting for sex, age, diagnostic category and medication status in the total sample, and then in each diagnostic group.

**Results:**

In the total sample, carriers of the risk allele had increased activation in the left amygdala. Group-wise analyses showed that this effect was significant in the BD group, but not in the other diagnostic groups. However, there was no significant interaction effect for the risk allele between BD and the other groups.

**Conclusions:**

These results indicate that *CACNA1C* SNP rs1006737 affects amygdala activity during emotional processing across all diagnostic groups. The current findings add to the growing body of knowledge of the pleiotropic effect of this polymorphism, and further support that ion channel dysregulation is involved in the underlying mechanisms of BD and SZ.

## Introduction

Despite the high heritability estimates for bipolar disorder (BD), the molecular genetic and neurobiological mechanisms for this disorder remain poorly understood. However, recent genome-wide association (GWA) studies with substantially more statistical power than former psychiatric genetic studies have provided evidence for new and promising candidate genes [1]. One of the most consistent findings is related to *CACNA1C* (calcium channel, voltage-dependent, L type, alpha 1C subunit) gene variants. The *CACNA1C* SNP rs1006737 was first found to be associated with BD in a combined analysis of two GWA study datasets (P = 3.15×10^−6^) [2]. When adding a third sample, this *CACNA1C* SNP was significantly associated with BD in the combined sample of 4,387 BD cases and 6,209 healthy controls (P = 7.0×10^−8^, OR = 1.181) [3]. Subsequent studies also reported association between *CACNA1C* and schizophrenia (SZ) [4], [5] and major depressive disorder (MDD) [4], [6], thereby supporting the hypothesis of genetic overlap between these severe psychiatric disorders.

*CACNA1C* is located on chromosome 10 and encodes an alpha-1 subunit of a voltage-dependent calcium (Ca_v_1.2) channel. Ca_v_1.2 channels can be found in cardiac smooth muscle, neuronal and endocrine cells, where they have a variety of functions including excitation-contraction coupling, endocrine secretion and regulation of neuronal Ca^2+^ transients, enzyme activity and transcription [7], [8]. Mutations in *CACNA1C* have been found to lead to Timothy syndrome, a lethal disorder consisting of somatic symptoms like cardiac arrhythmia, and psychiatric symptoms like autism and cognitive disability [9].

Emotional dysregulation is part of the clinical phenotype of BD and SZ, manifesting as mood swings as well as affect flattening. Morphometric studies point to structural abnormalities within the medial temporal lobe, which may cause altered responsivity to emotional stimuli among these patients. In particular, the amygdala has been extensively studied in BD. In a recent meta-analysis of 321 patients with bipolar I disorder and 442 healthy controls, amygdala volume was found to be greater in patients treated with lithium compared to controls and patients not treated with lithium [10]. A meta-analysis comprising 65 fMRI studies of 1074 healthy volunteers and 1040 BD cases, found evidence for amygdala over-activation in euthymic BD patients compared with healthy controls [11].

With regards to the potential involvement of BD risk genes in limbic system dysregulation, there are reports of an association between *CACNA1C* SNP rs1006737 and amygdala activity. In a study of 64 healthy individuals, carriers of the risk allele (AA/AG) had increased activity in the right amygdala in a monetary reward paradigm [12]. Another group reported enhanced activity in AA/AG individuals compared to GG individuals in the right amygdala during a fearful faces paradigm (N = 41 BD patients, 25 relatives and 50 healthy controls) [13]. A third study found healthy controls with the AA genotype to have trend significantly greater right amygdala activity than those with AG/GG in a negative faces matching paradigm [14]. Thus, there is some evidence supporting the hypothesis that *CACNA1C* SNP rs1006737 affects amygdala activity during different paradigms related to limbic system functioning.

Interestingly, two recent meta-analyses reported that *CACNA1C* was one out of three common genes for BD and SZ [1], [15]. These findings on the molecular genetic level are consistent with similarities in clinical and cognitive characteristics [16], and the continuum hypothesis for severe psychiatric disorders [17]. However, to the best of our knowledge *CACNA1C* has not been investigated with fMRI in individuals suffering from SZ. But a recent study of healthy controls found increased activity in the prefrontal cortex during executive cognition in *CACNA1C* risk allele carriers, a finding which could imply inefficient prefrontal functioning as a genetically conditioned mechanism underlying SZ [14].

Taken together, it remains unclear whether the effect of this gene on amygdala activity during emotional processing is general or confined to one or more diagnostic categories.

The primary aim of the current study was to test for altered amygdala activity in carriers of the *CACNA1C* SNP rs1006737 risk allele. Secondarily, we aimed to determine the specificity of such associations, by testing for potential differences between healthy controls and patients with BD or SZ. Therefore, we measured fMRI amygdala blood-oxygen-level dependence (BOLD) responses during a faces matching paradigm [18] in genotyped BD and SZ cases, as well as healthy controls.

## Materials and Methods

### Sample characteristics

The total number of individuals in this study was 250, including 66 BD cases, 61 SZ cases and 123 healthy control subjects. All participants were of Northern European origin (96% were born in Norway with Norwegian parents, 4% had one or both parents from another Northern European country), part of the ongoing TOP (Thematic Organized Psychosis) Study, and included from 2003 to 2009.

To be included in the study, patients had to be between 18 and 65 years, have a DSM-IV diagnosis of a bipolar spectrum or schizophrenia spectrum disorder, and be willing and able to provide written informed consent. Exclusion criteria were an IQ score below 70 and reporting a history of head injury or neurological disorder. In the healthy control group, we also excluded subjects if they or their close relatives had a lifetime history of a severe psychiatric disorder (SZ, BD and major depression). Subjects with a history of a medical condition potentially interfering with brain function (hypothyroidism, uncontrolled hypertension and diabetes), and an illicit drug abuse/addiction diagnosis were also excluded.

Patients were recruited from psychiatric in- and out-patient hospital units in the Oslo area, and had been diagnosed with bipolar I disorder (N = 30), bipolar II disorder (N = 32), bipolar disorder not otherwise specified (N = 4), schizophrenia (N = 48), schizoaffective disorder (N = 9) or schizophreniform disorder (N = 4), according to DSM-IV using the Structural Clinical Interview for DSM-IV (SCID) [19]. 23 out of 30 (76.7%) patients with bipolar I disorder had a SCID-verified lifetime history of psychosis, and the corresponding numbers were 3 out of 32 (9.4%) for bipolar II disorder and 2 out of 4 (50%) for bipolar disorder not otherwise specified. Diagnostic evaluation was performed by trained psychologists and psychiatrists, of whom all participated regularly in diagnostic meetings supervised by professors in psychiatry. Reliability measures of the diagnostic assessment in the TOP study were performed, and the overall agreement for the DSM-IV diagnostic categories tested was 82% and the overall Kappa 0.77 (95% CI: 0.60–0.94). Information on education, age of onset, number of relapses, medication status, alcohol and illegal substance abuse was obtained during an initial clinical interview. A three-hour neuropsychological test battery, including Wechsler Abbreviated Scale of Intelligence (WASI), was carried out by trained clinical psychologists [16].

On the day of scanning, patients underwent an abbreviated re-interview including Young Mania Rating Scale (YMRS) [20], Inventory of Depressive Symptoms (IDS) [21] and Positive and Negative Syndrome Scale (PANSS) [22]. For patients lacking data for this re-interview, we used corresponding data from the clinical interview.

The healthy control subjects were randomly recruited from the same catchment area as the patients, and underwent an initial interview where demographic and clinical information was obtained. Clinical assessment of the patients and healthy controls participating in the TOP study is described in details in a previous report [23].

Carriers of the risk allele did not differ significantly from the GG homozygotes with respect to demographical variables within the total sample or any of the diagnostic groups (Table S1). Further, the clinical characteristics did not differ between genotype groups, except that risk allele carriers in the total patient sample had significantly lower Global Assessment of Functioning-symptom score (GAF-S) than those with the GG genotype (P = 0.01). However, this was not seen in the subgroups, and was probably due to a higher frequency of the risk allele in the SZ group (43/61 (70.5%)) compared to the BD group (34/66 (51.5%)). For further details on demographic and clinical characteristics, see Table S1.

### Ethics Statement

The Norwegian Scientific-Ethical Committees and the Norwegian Data Protection Agency approved the study. All subjects have given written informed consent prior to inclusion into the project.

### Genotyping

Genomic DNA was extracted from whole blood. *CACNA1C* SNP rs1006737 was genotyped in the 250 subjects participating in this study using Affymetrix Gene Chip Genome-Wide SNP 6.0 array (AffymetrixInc, Santa Clara, CA, USA), as described in details elsewhere [24], [25]. There was no deviation from Hardy–Weinberg equilibrium (HWE) in the controls (P = 0.84) or in the cases (P = 0.57), using PLINK (version 1.07;http://pngu.mgh.harvard.edu/purcell/plink/) [26].

### Experimental paradigm

A widely used and validated paradigm was employed to elicit amygdala reactivity [18]. In this task participants select which of two stimuli (displayed at the bottom of the screen) matches a target stimulus (displayed at the top). The images displayed were either human faces expressing anger or fear (faces matching task) or geometrical shapes (the sensorimotor control task). Participants completed 4 blocks of the faces matching task, where each block consisted of 6 emotion-specific face trios derived from a standard set of facial affect pictures [27]. Interleaved between these blocks, participants completed 5 blocks of the sensorimotor control task. Each trial (faces or shapes) was presented for 5.4 seconds with no inter-stimulus interval, for a total block length of 32.6 seconds. The total paradigm lasted 310 seconds. E-prime software (version 1.0 Psychology Software Tools, Inc, Pittsburgh, PA, USA) controlled the presentations of the stimuli using VisualSystem (NordicNeuroLab, Bergen, Norway). Response times and accuracy were recorded through MR-compatible ResponseGrips (NordicNeuroLab, Bergen, Norway). Behavioural data was missing for 13 individuals.

### Image acquisition

MRI scans were acquired on a 1.5 T Siemens Magnetom Sonata scanner (Siemens Medical Solutions, Erlangen, Germany) supplied with a standard head coil. Volumes (n = 152, 24 axial slices, 4 mm thick with 1 mm gap) covering the whole brain were acquired in the axial plane, using a BOLD EPI sequence (TR = 2040 ms, TE = 50 ms, flip angle = 90°, matrix 64×64, FOV 192×192 mm). The first seven volumes were discarded. Prior to BOLD fMRI scanning, a sagittal T1-weighted 3D Magnetization Prepared Rapid Gradient Echo (MPRAGE) scan (TR = 2000 ms, TE = 3.9 ms, flip angle = 7°, matrix 128×128, FOV 256×256 mm) was collected for better localization of functional data.

### fMRI data analyses

All fMRI volumes were preprocessed and analysed with Statistical Parametric Mapping (SPM2) (http://www.fil.ion.ucl.ac.uk/spm) implemented in MATLAB7.1 (The Mathworks Inc, Natick, Massachusetts). All of the functional images were realigned to the first image in the time series to correct for head motion [28]. All subjects moved less than 2.5 mm in any direction during the scan. Subsequently, the mean functional image and the anatomical image were coregistered to ensure that they were aligned. The images were spatially normalized to the stereotactical MNI template [28], and resampled at 2×2×2 mm voxels. The images were smoothed using a 6 mm full width-half maximum (FWHM) isotropic kernel. Data were high-pass filtered using a cutoff value of 128 s. The fMRI data for all subjects were first analysed using a single-subject fixed-effect model. The model was built by convolving boxcar functions for the onsets of the two different conditions (faces and figures) with a canonical hemodynamic response function (HRF). Individual contrast images were created by subtracting “figures” from “faces”. The contrast images for faces versus figures for each subject were entered into a random effects statistical model. These data were analysed with a region of interest (ROI) approach and a pre-defined anatomical mask (bilateral amygdala) derived from the Wake Forest University PickAtlas for SPM2 [29]. The six movement parameters were included in the first-level analyses as regressors without interest.

### Statistical analysis

For the overall sample, we used an ANCOVA model comparing amygdala activity in risk-allele carriers (AA/AG) with the corresponding activity in carriers of the protective allele (GG), using sex, age, diagnostic category (BD, SZ, healthy control) and medication status as covariates. Medication status was dichotomised for each of the categories Antipsychotics, Lithium, Antidepressants, Anticonvulsives and Hypnotics. We tested for potential specific effects in each diagnostic group (BD, SZ and healthy controls) and phenotypic subcategory (bipolar I disorder, bipolar II disorder, psychotic and non-psychotic features within the BD group, schizophrenia patients within the SZ group), for genotype×diagnosis interactions, and for effect of diagnostic category on amygdala activity. Findings were regarded as significant if they obtained a small volume corrected Family-Wise Error (FWE) P-value below 0.05 within the respective ROI (amygdala).

Statistical analyses of behavioural, sociodemographic and clinical data were performed with IBM SPSS Statistics version 19.0.

## Results

### Behavioural results

Genotype group and diagnosis did not influence the accuracy rate significantly. The mean response time (RT) was significantly longer for individuals in the BD group (RT = 1255 milliseconds (ms)) and SZ group (RT = 1231 ms) than those in the healthy control group (RT = 1065 ms) (P<0.001), but did not differ significantly with respect to genotype group. For details, see Table S1.

### fMRI results

**Figure 1:**
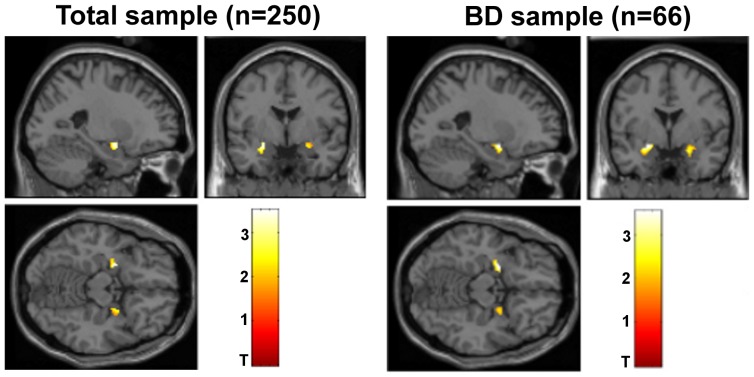
Increased amygdala activity in *CACNA1C* SNP rs1006737 risk allele carriers. Carriers of the *CACNA1C* SNP rs1006737 risk allele A have significantly increased activity in the left amygdala in the total sample (x = −24, y = −2, z = −14; FWE P = 0.026) and BD subgroup (x = −24, y = 0, z = −14; FWE P = 0.041), and non-significantly increased activity in the right amygdala in the total sample (x = 26, y = 0, z = −16) and BD subgroup (x = 22, y = 0, z = −20) compared with GG homozygotes during a negative faces paradigm. Abbreviations: BD, bipolar disorder; FWE, family-wise error. Threshold for significance in Figure 1 is set to Nominal P<0.05 within the ROI.

The main fMRI results are presented in Table 1 and Figure 1. In the total sample (N = 250), carriers of the *CACNA1C* SNP rs1006737 risk allele (AA/AG) showed significantly increased activation in the left amygdala (x = −24, y = −2, z = −14; Z = 3.47; cluster-size = 72), with an FWE-corrected P-value of 0.026. The risk allele was also significantly associated with enhanced activity in the left amygdala (x = −24, y = 0, z = −14; Z = 3.35; cluster-size = 91) in the BD group (FWE-corrected P = 0.041). There were also AA/AG associated increased activations in the right amygdala in the total sample (x = 26, y = 0, z = −16, Z = 2.65, cluster-size = 32) and in the BD group (x = 22, y = 0, z = −20, Z = 2.54, cluster-size = 61), but these did not reach FWE-corrected significance level. There were no FWE-corrected significant findings in SZ individuals or healthy controls, but the activation patterns had the same direction in all diagnostic groups for the most significant voxel in the total sample (Figure S1), and there were only nominally significant results in amygdala for the AA/AG>GG contrast and none for the opposite contrast (GG>AA/AG) in any of the groups (Table 1). There was no significant genotype×diagnosis interaction effect between BD and the other groups. Furthermore, there was no significant effect of diagnosis or medication status on amygdala activity. Carriers of the rs1006737 risk variant did not reach FWE-corrected significance level in any of the phenotypic subcategories, and there was no significant interaction effect of the risk allele between bipolar I and II disorder or between psychotic and non-psychotic BD subjects (Table S2 and Table S3).

**Table 1:** Results for *CACNA1C* SNP rs1006737 (AA+AG>GG) effect on amygdala activation in a sample of bipolar disorder and schizophrenia cases and healthy controls.

Hemisphere	Group	x	y	z	Cluster size	Z	P uncorrected	P FWE corrected
Left	Total sample	−24	−2	−14	72	3.47	0.000	0.026
	BD	−24	0	−14	91	3.35	0.000	0.041
	SZ	–	–	–	–	–	n.s.	–
	CTR	−24	−2	−12	16	2.51	0.006	0.284
Right	Total sample	26	0	−16	32	2.65	0.004	0.217
	BD	22	0	−20	61	2.54	0.006	0.278
	SZ	24	−4	−16	26	2.24	0.013	0.449
	CTR	28	4	−16	2	1.96	0.025	0.598

Abbreviations: BD, bipolar disorder; SZ, schizophrenia; CTR, healthy controls; FWE, Family-wise error rate; n.s., non-significant.

Only nominally significant results (Nominal P = <0.05) are shown.

## Discussion

The main finding in this study was an enhanced amygdala activation in carriers of the *CACNA1C* SNP rs1006737 risk allele. This is in line with two previous studies [12], [13]. One of these studies found that individuals with the risk allele have increased activity in the right amygdala during a reward paradigm (N = 64) [12], and the other reported increased activity in the right amygdala during a fear-face paradigm (N = 116) [13]. In the current study, we found significantly enhanced activation in the left amygdala during a negative faces paradigm. But there was also nominally significantly increased activity in the right amygdala in our sample, although this finding did not remain significant after FWE-correction (Table 1).

As increased amygdala activity in *CACNA1C* risk allele carriers has been reported for both reward and aversive paradigms, it is uncertain whether these responses reflect different psychological responses and neurobiological pathways. These findings might also represent a general effect, irrespective of emotional valence. The latter interpretation is in accordance with recent data suggesting that amygdala is occupied with relevance detection in general, rather than with only fear-related or reward-related information [30], [31].

In the present study we found increased activity in the left amygdala, while two other studies reported increased activity in the right amygdala [12], [13]. With regards to the laterality of enhanced amygdala activity in BD, a meta-analysis reported evidence for this effect being most pronounced in the left hemisphere [11], which is in line with our findings. But more and larger studies are needed to address the question of amygdala laterality in BD. Further, despite several hypotheses on amygdala laterality in general, it is still unclear whether the left and right amygdalae are involved in different psychological mechanisms [32].

When performing group-wise analyses, we found an increased amygdala activity in risk allele carriers within the BD group, but not in the SZ or healthy control group. However, there was no significant diagnosis×genotype interaction, a phenomenon that was probably due to the same direction of the activation pattern through all diagnostic groups (Figure S1). Further, there were no interaction effects for the risk allele between the phenotypic subcategories within the BD spectrum (Table S2). Thus, we can not conclude with diagnostic specificity for the current effect of this polymorphism.

Interestingly, recent GWA studies found the presently investigated SNP to be significantly associated with BD (P = 1.7×10^−5^; OR = 1.11) as well as SZ (P = 1.2×10^−6^; OR = 1.11) [1], [15]. This suggests susceptibility of similar effect sizes on the clinical phenotype level, a finding which is in accordance with the current lack of evidence for specificity at the brain activity level. Two recent studies might shed further light on the genotype/phenotype relationship between the *CACNA1C* polymorphism and BD and SZ. One of these studies found that healthy risk allele carriers had elevated hippocampus activity during emotional processing, and increased activity in the prefrontal cortex during executive cognition [14]. As the former phenomenon has been reported in BD and the latter in SZ, these results correspond to the findings of a pleiotropic effect of this SNP. Another recent study reported impaired working memory in risk allele carriers among SZ cases and healthy controls, but not in BD cases, potentially implying differential specificity in different diagnostic groups [33] for this neurocognitive phenotype.

This phenomenon of unspecific effects at some levels and differential diagnostic specificity at other levels could be explained by different, although overlapping, overall genetic architecture between BD and SZ patients [34]. In such a model, the cumulative effect of all risk variants, including gene-gene and gene-environment interactions, could condition the role of the current *CACNA1C* SNP in the pathophysiological processes related to these disorders.

Genetic variations in *CACNA1C* have been shown to imply additional psychiatric manifestations to those observed in BD and SZ. Patients with the above-mentioned Timothy syndrome are characterized by symptoms like autism and cognitive disability. In a proposed model of a spectrum of psychiatric disorders with autism in the neurodevelopmental end and MDD in the affective end [17], it is possible that polymorphisms in or around the *CACNA1C* gene could increase the risk of developing less severe conditions than those observed in Timothy syndrome, like BD and SZ. This is further supported by the fact that rs1006737 is situated in one of the introns of *CACNA1C*, thus probably affecting pathophysiological pathways related to these disorders by regulating the expression of the protein, and not by altering the structure, as is the case with the de novo mutations in Timothy syndrome, which are located in one of the exons [9]. In this respect, it is noteworthy that a recent post mortem brain expression study found healthy carriers of the *CACNA1C* risk-associated SNP to express higher levels of *CACNA1C* mRNA than carriers of the protective allele [14].

Hence, genetically conditioned calcium channel dysregulation resulting from *CACNA1C* risk variants might be a common mechanism increasing the risk for developing several neuropsychiatric disorders, by affecting various brain structures, including amygdala.

Taken together, the current findings provide evidence that the *CACNA1C* SNP rs1006737 is associated with increased amygdala activity across different diagnostic groups. These findings add to the growing body of knowledge on the pleiotropic effect of this polymorphism.

## Supporting Information

Table S1 **Demographic data and clinical characterization for individuals genotyped for rs1006737 and participating in a negative faces functional MRI study.** Abbreviations: BD, bipolar disorder; SZ, schizophrenia; CTR, controls; SD, standard deviation; WASI, Wechsler Abbreviated Scale of Intelligence; IDS, Inventory of Depressive Symptoms; YMRS, Young Mania Rating Scale; PANSS, Positive and Negative Syndrome Scale; GAF-S, Global Assessment of Functioning–symptom score; GAF-F, Global Assessment of Functioning–function score; ms, milliseconds. aMean age at fMRI scanning. bLast six months.
(DOC)

Table S2 **Results for the effect of** ***CACNA1C*** **SNP rs1006737 (AA+AG>GG) interaction with diagnostic category on amygdala activation.** Abbreviations: BD, bipolar disorder; SZ, schizophrenia; CTR, healthy controls; BDI, bipolar I disorder; BDII, bipolar II disorder; FWE, Family-wise error rate; n.s., non-significant. Only nominally significant results (Nominal P = <0.05) are shown.
(DOC)

Table S3 **Results for** ***CACNA1C*** **SNP rs1006737 (AA+AG>GG) effect on amygdala activation in phenotypic subcategories.** Abbreviations: BD, bipolar disorder; BDI, bipolar I disorder; BDII, bipolar II disorder; FWE, Family-wise error rate; n.s., non-significant. Only nominally significant results (Nominal P = <0.05) are shown.
(DOC)

Figure S1 **Parameter estimates for diagnostic and genotype groups for** ***CACNA1C*** **SNP rs1006737 effect on amygdala activation.** Parameter estimates for the contrast AA+AG>GG in the voxel with highest activation (x = −24, y = −2, z = −14) in the total sample (N = 250) in different diagnostic and genotype groups. Prot = GG genotype group. Risk = AA+AG genotype group. Abbreviations: BD, bipolar disorder; SZ, schizophrenia; CTR, healthy controls.
(DOC)
