# Correction: *CACNA1C* Risk Variant and Amygdala Activity in Bipolar Disorder, Schizophrenia and Healthy Controls

**DOI:** 10.1371/annotation/3b96507c-056e-4317-8234-de84b2b8113b

**Published:** 2013-11-06

**Authors:** Martin Tesli, Kristina C. Skatun, Olga Therese Ousdal, Andrew Anand Brown, Christian Thoresen, Ingrid Agartz, Ingrid Melle, Srdjan Djurovic, Jimmy Jensen, Ole A. Andreassen

The Supporting Information Table 1 was incorrectly uploaded. Please view the correct Supporting Information Table 1 file here: 
