# Supplementary figures and images for: CACNA1C Risk Variant and Amygdala Activity in Bipolar Disorder, Schizophrenia and Healthy Controls
Source: PLoS One. 2013 Feb 20;8(2):e56970. doi: 10.1371/journal.pone.0056970 (PMC3577650; doi:10.1371/journal.pone.0056970)

**
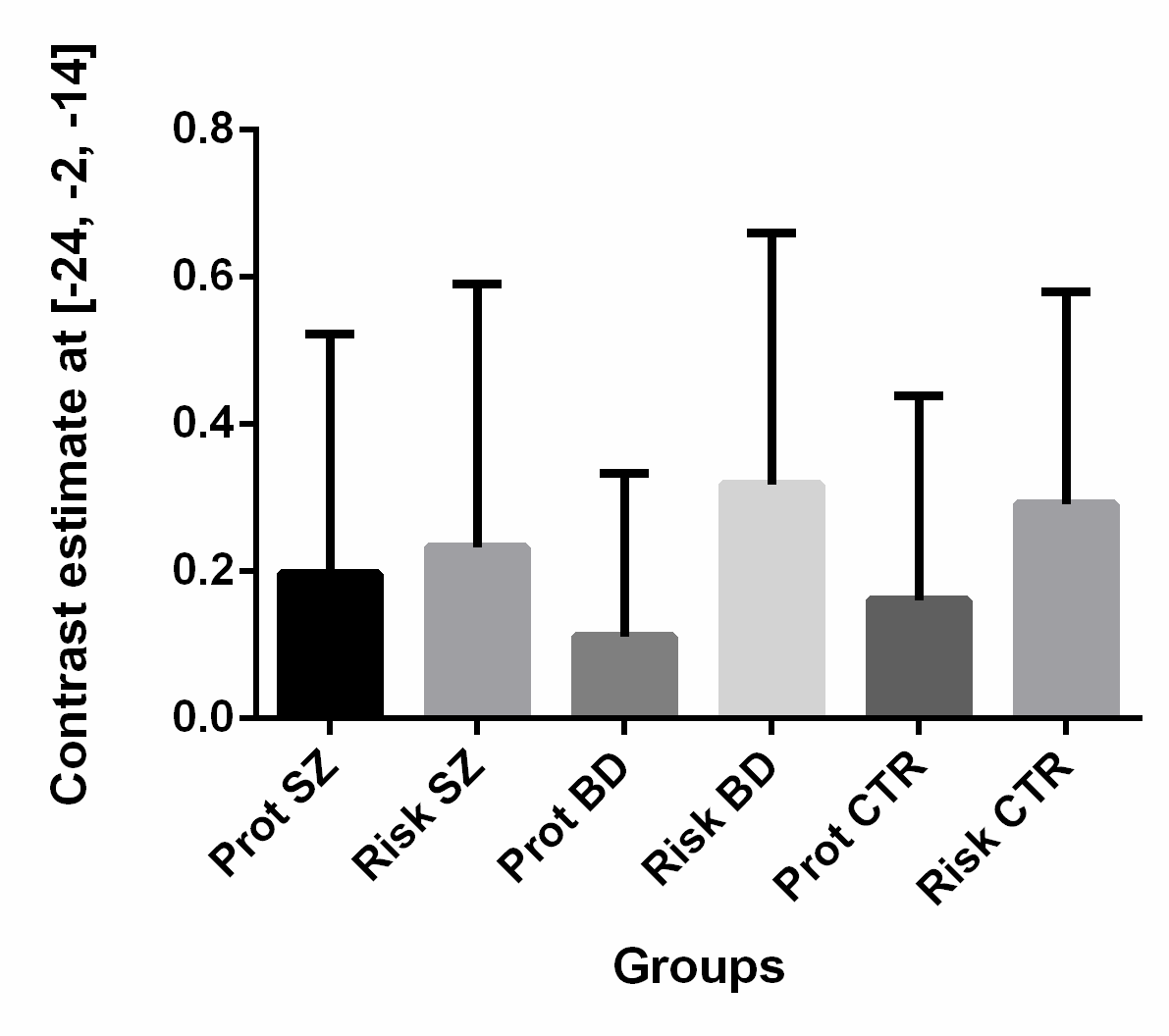
**

Supplement: Table S1 — Demographic data and clinical characterization for individuals genotyped for rs1006737 and participating in a negative faces functional MRI study. Abbreviations: BD, bipolar disorder; SZ, schizophrenia; CTR, controls; SD, standard deviation; WASI, Wechsler Abbreviated Scale of Intelligence; IDS, Inventory of Depressive Symptoms; YMRS, Young Mania Rating Scale; PANSS, Positive and Negative Syndrome Scale; GAF-S, Global Assessment of Functioning–symptom score; GAF-F, Global Assessment of Functioning–function score; ms, milliseconds. aMean age at fMRI scanning. bLast six months. (DOC) [file pone.0056970.s001.doc]
