# Supplementary material for: CACNA1C Risk Variant and Amygdala Activity in Bipolar Disorder, Schizophrenia and Healthy Controls
Source: PLoS One. 2013 Feb 20;8(2):e56970. doi: 10.1371/journal.pone.0056970 (PMC3577650; doi:10.1371/journal.pone.0056970)
Supplement: Supplementary file 2 [file pone.0056970.s002.doc]

Hemisphere	Group	x	y	z	Cluster size	Z	P uncorrected	P FWE corrected
Left	BD vs CTR	–	–	–	–	–	n.s.	–
	BD vs SZ	–	–	–	–	–	n.s.	–
	BDI vs BDII	–	–	–	–	–	n.s.	–
	BDII vs BDI	-30	0	-22	2	2.02	0.022	0.578
	BDII vs BDI	-30	0	-26	1	1.84	0.033	0.678
	Psychotic vs non-psychotic BD	–	–	–	–	–	n.s.	–
	Non-psychotic vs psychotic BD	-30	-2	-28	4	2.60	0.005	0.247
Right	BD vs CTR	–	–	–	–	–	n.s.	–
	BD vs SZ	32	-2	-12	1	1.71	0.043	0.734
	BDI vs BDII	–	–	–	–	–	n.s.	–
	BDII vs BDI	–	–	–	–	–	n.s.	–
	Psychotic vs non-psychotic BD	–	–	–	–	–	n.s.	–
	Non-psychotic vs psychotic BD	34	2	-24	18	2.14	0.016	0.505
	Non-psychotic vs psychotic BD	26	-2	-28	3	1.94	0.026	0.623
