# Supplementary material for: CACNA1C Risk Variant and Amygdala Activity in Bipolar Disorder, Schizophrenia and Healthy Controls
Source: PLoS One. 2013 Feb 20;8(2):e56970. doi: 10.1371/journal.pone.0056970 (PMC3577650; doi:10.1371/journal.pone.0056970)
Supplement: Supplementary file 3 [file pone.0056970.s003.doc]

Hemisphere	Group	x	y	z	Cluster size	Z	P uncorrected	P FWE corrected
Left	BDI	-22	0	-12	7	2.07	0.019	0.607
	BDII	-26	-2	-12	14	2.24	0.013	0.478
	Psychotic BD	-24	0	-14	49	2.71	0.003	0.198
	Non-psychotic BD	-26	-4	-14	17	2.07	0.019	0.546
	Schizophrenia	–	–	–	–	–	n.s.	–
Right	BDI	–	–	–	–	–	n.s.	–
	BDII	24	4	-20	7	2.10	0.018	0.557
	BDII	26	-8	12	1	1.67	0.047	0.777
	Psychotic BD	22	0	-18	5	1.86	0.031	0.666
	Non-psychotic BD	24	0	-24	10	1.81	0.035	0.691
	Schizophrenia	24	-4	16	32	2.10	0.018	0.539
