# Supplementary material for: Correction: CACNA1C Risk Variant and Amygdala Activity in Bipolar Disorder, Schizophrenia and Healthy Controls
Source: PLoS One. 2013 Nov 6;8(11):10.1371/annotation/3b96507c-056e-4317-8234-de84b2b8113b. doi: 10.1371/annotation/3b96507c-056e-4317-8234-de84b2b8113b (PMC3819414; doi:10.1371/annotation/3b96507c-056e-4317-8234-de84b2b8113b)
Supplement: Supplementary file 1 [file pone.3b96507c-056e-4317-8234-de84b2b8113b.s001.doc]

	Clinical group				Genotype			Clinical group x genotype								
	BD	SZ	CTR		AA+AG	GG		BD			SZ			CTR		
				P			P	AA+AG	GG	P	AA+AG	GG	P	AA+AG	GG	P
N (Females, %)	66 (57.6)	61 (36.1)	123 (43.9)	0.05	148 (43.2)	102 (49.0)	0.37	34 (52.9)	32 (62.5)	0.43	43 (34.9)	18 (38.9)	0.77	71 (43.7)	52 (44.2)	0.95
Mean age (SD)a	34.8 (10.6)	33.7 (8.6)	34.6 (9.0)	0.77	33.9 (8.5)	35.2 (10.4)	0.30	34.2 (10.0)	35.5 (11.4)	0.62	33.5 (8.5)	34.1 (9.0)	0.81	34.0 (7.8)	35.3 (10.4)	0.43
Education (years), mean (SD)	14.1 (3.0)	13.7 (2.5)	14.3 (2.3)	0.32	14.2 (2.5)	13.9 (2.7)	0.32	13.9 (3.0)	14.4 (3.0)	0.52	14.0 (2.4)	13.1 (2.4)	0.21	14.6 (2.1)	13.9 (2.5)	0.12
WASI, mean (SD)	109.4 (11.5)	108.2 (13.7)	115.9 (10.0)	0.00	110.9 (12.6)	113.4 (10.3)	0.10	107.4 (12.7)	111.7 (9.7)	0.14	107.7 (14.1)	109.5 (13.2)	0.64	114.5 (10.6)	115.8 (9.2)	0.46
IDS, mean (SD)	15.4 (13.1)	11.7 (11.5)		0.10	13.2 (12.4)	14.2 (12.6)	0.64	13.8 (12.4)	17.0 (13.9)	0.33	12.7 (12.6)	9.4 (8.4)	0.32			
YMRS, mean (SD)	1.9 (3.5)	1.4 (4.0)		0.45	1.6 (3.7)	1.8 (3.9)	0.72	1.4 (1.9)	2.4 (4.6)	0.27	1.7 (4.6)	0.8 (1.6)	0.43			
PANSS total positive score, mean (SD)	9.5 (3.3)	13.0 (6.1)		0.00	11.8 (5.7)	10.2 (4.1)	0.10	9.5 (3.6)	9.5 (3.0)	0.96	13.6 (6.4)	11.4 (5.4)	0.22			
GAF-S, mean (SD)	58.2 (9.5)	42.6 (10.6)		0.00	48.5 (13.0)	54.2 (11.2)	0.01	57.4 (10.1)	59.0 (8.9)	0.49	41.5 (10.7)	45.7 (9.5)	0.16			
GAF-F, mean (SD)	55.3 (12.2)	44.4 (9.9)		0.00	49.0 (13.0)	51.7 (11.2)	0.23	55.3 (13.3)	55.4 (11.2)	1.00	44.0 (10.5)	45.2 (7.9)	0.67			
Age of onset, mean (SD)	21.2 (8.6)	24.8 (7.8)		0.02	23.3 (8.2)	22.4 (8.6)	0.57	20.9 (8.5)	21.7 (8.7)	0.71	25.2 (7.5)	23.8 (8.5)	0.53			
Duration of illness, mean (SD)	18.1 (10.3)	13.1 (6.2)		0.00	14.9 (8.4)	17.1 (9.5)	0.17	17.8 (9.9)	18.4 (10.9)	0.79	12.6 (6.3)	14.7 (5.8)	0.24			
No. of depressive episodes, mean (SD)	7.3 (12.9)	2.3 (6.0)		0.01	5.4 (12.7)	4.1 (5.3)	0.53	8.9 (17.2)	5.7 (5.8)	0.33	2.7 (6.9)	0.9 (0.9)	0.32			
No. of manic episodes, mean (SD)	1.5 (2.8)	0.1 (0.3)		0.00	0.6 (1.5)	1.2 (2.9)	0.15	1.2 (2.0)	1.8 (3.5)	0.86	0.1 (0.4)	0.1 (0.2)	0.68			
No. of hypomanic episodes, mean (SD)	14.0 (38.0)	0.0 (0.1)		0.01	5.8 (19.3)	9.6 (38.3)	0.47	13.2 (27.6)	14.8 (47.1)	0.40	0.0 (0.2)	0.0 (0.0)	0.53			
Alcohol abuseb, n (%)	8 (12.0)	5 (8.2)		0.47	10 (13.2)	3 (6.0)	0.20	6 (17.6)	2 (6.3)	0.16	4 (9.3)	1 (5.6)	0.63			
Use of illegal substancesb, n (%)	4 (6.1)	7 (11.5)		0.28	7 (9.2)	4 (8.0)	0.82	3 (8.8)	1 (3.1)	0.33	4 (9.3)	3 (16.7)	0.41			
Accuracy rate, faces (%)	98.1 (10.6)	97.9 (8.4)	99.2 (29.3)	0.25	98.7 (7.3)	98.5 (6.7)	0.86	96.8 (14.5)	99.6 (12.9)	0.32	98.8 (31.6)	95.2 (15.5)	0.17	99.4 (19.0)	98.8 (39.3)	0.24
